# In vivo and ex vivo analyses of amyloid toxicity in the Tc1 mouse model of Down syndrome

**DOI:** 10.1177/0269881117743484

**Published:** 2017-12-07

**Authors:** Gaëlle Naert, Valentine Ferré, Emeline Keller, Amy Slender, Dorota Gibbins, Elizabeth MC Fisher, Victor LJ Tybulewicz, Tangui Maurice

**Affiliations:** 1 INSERM U1198, Montpellier, France; 2 University of Montpellier, Montpellier, France; 3 EPHE, Paris, France; 4 Francis Crick Institute, London, UK; 5 Institute of Neurology, University College London, London, UK; 6 Imperial College, London, UK

**Keywords:** Alzheimer’s disease, Tc1 mice, tyrosine phosphorylation regulated kinase 1A gene, amyloid-β [25-35] peptide, memory

## Abstract

**Rationale:**

The prevalence of Alzheimer’s disease is increased in people with Down syndrome. The pathology appears much earlier than in the general population, suggesting a predisposition to develop Alzheimer’s disease. Down syndrome results from trisomy of human chromosome 21, leading to overexpression of possible Alzheimer’s disease candidate genes, such as amyloid precursor protein gene. To better understand how the Down syndrome context results in increased vulnerability to Alzheimer’s disease, we analysed amyloid-β [25-35] peptide toxicity in the Tc1 mouse model of Down syndrome, in which ~75% of protein coding genes are functionally trisomic but, importantly, not amyloid precursor protein.

**Results:**

Intracerebroventricular injection of oligomeric amyloid-β [25-35] peptide in three-month-old wildtype mice induced learning deficits, oxidative stress, synaptic marker alterations, activation of glycogen synthase kinase-3β, inhibition of protein kinase B (AKT), and apoptotic pathways as compared to scrambled peptide-treated wildtype mice. Scrambled peptide-treated Tc1 mice presented high levels of toxicity markers as compared to wildtype mice. Amyloid-β [25-35] peptide injection in Tc1 mice induced significant learning deficits and enhanced glycogen synthase kinase-3β activity in the cortex and expression of apoptotic markers in the hippocampus and cortex. Interestingly, several markers, including oxidative stress, synaptic markers, glycogen synthase kinase-3β activity in the hippocampus and AKT activity in the hippocampus and cortex, were unaffected by amyloid-β [25-35] peptide injection in Tc1 mice.

**Conclusions:**

Tc1 mice present several toxicity markers similar to those observed in amyloid-β [25-35] peptide-treated wildtype mice, suggesting that developmental modifications in these mice modify their response to amyloid peptide. However, amyloid toxicity led to severe memory deficits in this Down syndrome mouse model.

## Introduction

Down syndrome (DS), which is caused by the presence of three complete or partial copies of chromosome 21 (Hsa21) is the most common chromosomal disorder and is also the most common genetic cause of cognitive impairment. The increased protein expression of genes on Hsa21 leads to a cascade of effects in the foetal and post-natal development of brain structure and subsequent biochemical and behavioural effects through the lifespan of DS subjects (de la Torre and Dierssen, 2012). Among the ~232 coding genes of chromosome 21, superoxide dismutase (*SOD1*), β-secretase, 2 (*BACE2*), S100 calcium binding protein B (*S100β*), dual specificity tyrosine phosphorylation regulated kinase 1A (*DYRK1A*), and amyloid precursor protein (*APP*) are located in Hsa21 (Choong et al., 2015; Rachidi and Lopes, 2008). Triplication of *APP* is thought to largely contribute to the onset of Alzheimer’s disease (AD) in people with DS, who tend to develop AD, with a higher prevalence at a much younger age than the euploid population (de la Torre and Dierssen, 2012). DS life expectancy has greatly increased in the last decades and hence incidence of AD has increased in this population. AD rates as high as 75% may occur in DS between ages of 60–69 years, as compared with only 14% in the general population at an age of 65 years (de la Torre and Dierssen, 2012). Indeed, by the age of 40 years, individuals with DS invariably develop amyloid plaques and neurofibrillary tangles (NFTs), which are the major hallmarks of AD in addition to the progressive decline in executive and cognitive functions (Lott and Head, 2005; Selkoe, 2002).

Senile plaques formed by aggregation of Aβ peptides, the proteolytic cleavage product of APP by β- and γ-secretases, are accompanied by an important inflammatory reaction around dystrophic neuritis, with activated microglia and reactive astrocytes (Haass and Selkoe, 2007; Selkoe, 2002). NFTs result from Tau protein hyperphosphorylation and aggregation into paired helical filaments. As in AD cases in the general population, amyloid deposits in DS brains are associated with microglial and astroglial activations (Lott and Head, 2005). The earlier onset and increased prevalence of AD in DS could be explained in part by the triplication of Hsa21 genes increasing the risk of AD and promoting the production of Aβ and the aberrant phosphorylation of Tau, such as *SOD1, BACE2, S100β* or *DYRK1A*. Moreover, the trisomic Ts65Dn and Ts1Cje mouse models of DS exhibited aberrant phosphorylation of Tau (Liu et al., 2008; Shukkur et al., 2003).

Among the various kinases involved in AD, and particularly in Tau phosphorylation, glycogen synthase kinase-3β (GSK-3β) and cyclin-dependent kinase 5 (CDK-5) are the most implicated (Baumann et al., 1993; Flaherty et al., 2000; Sperber et al., 1995). Total GSK-3β expression and CDK5 expression are not affected in DS brains (Liu et al., 2008; Swatton et al., 2004). However, GSK-3β expression can be stimulated by DSCR1 (RCAN1), which appears to be another important gene located in the DS critical region. Therefore, DSCR1 overexpression might contribute to neurofibrillary degeneration in DS (Ermak et al., 2006). Moreover, DYRK1A has emerged as another kinase that also directly phosphorylates Tau and primes it for further phosphorylations by GSK-3β (Woods et al., 2001). In addition to other kinases, DYRK1A also participates in APP phosphorylation on Thr^668^ (Ryoo et al., 2008). It has been proposed that DYRK1A may favour the appearance of some pathological hallmarks of neurotoxicity by phosphorylating key proteins, such as APP, Tau, α-synuclein or presenilin-1 (Wegiel et al., 2011). As observed in DS brains, DYRK1A mRNA and protein levels are increased in brain of AD patients (Ferrer et al., 2005; Kimura et al., 2007). In addition, DYRK1A mRNA level was up-regulated along with an increase in the Aβ species level in the brain of Tg-PS1/APP transgenic mice (Kimura et al., 2007). This increase of DYRK1A expression could be attributed to Aβ, since Aβ treatment increased DYRK1A transcript levels in neuroblastoma cells (Kimura et al., 2007). Remarkably, Aβ has been reported to be elevated in transgenic mice overexpressing DYRK1A (Ryoo et al., 2008), suggesting the existence of a regulation loop between DYRK1A expression and Aβ production. Thus, the overexpression of DYRK1A in DS may link Aβ production with Tau phosphorylation, the two major hallmarks of AD neurotoxicity.

We have previously investigated DYRK1A in Aβ toxicity using the amyloid-β [25-35] (Aβ_25-35_) peptide mouse model (Naert et al., 2015). Using L41, a compound that inhibits DYRK1A kinase activity, we demonstrated that DYRK1A is involved in vivo in Aβ_25-35_ toxicity (Naert et al., 2015). As *DYRK1A* is one of the genes triplicated in DS and a major kinase involved in DS features, we hypothesised that Aβ_25-35_ toxicity would be affected in the context of DS. In this study, we used the unique transchromosomic Tc1 mouse, which carries a freely segregating Hsa21, in a background of a complete set of mouse chromosomes. Some deletions and rearrangements occurred during construction of the model, and, as a result, Tc1 mice are functionally trisomic for ~75% of protein coding genes (Choong et al., 2015; Gribble et al., 2013), but, importantly, not *APP*. Therefore Tc1 mice are a good model in which to test the contribution of Hsa21 genes other than APP to AD on a DS background. These mice do not show any spontaneous amyloid deposition (Ahmed et al., 2013; Gribble et al., 2013; O’Doherty et al., 2005). A robust increase of DYRK1A expression has been reported in brain of Tc1 mice, without any change in GSK-3β and CDK-5 kinases (Ahmed et al., 2013; Sheppard et al., 2012). We therefore compared the toxicity induced by intracerebroventricular (i.c.v.) injection of Aβ_25-35_ in young (2.5–3.5 months) WT and Tc1 mice. We report that cognitive impairments and toxicity induced by Aβ_25-35_ are modified in this mouse model of DS.

## Methods

### Animals

Tc1 mice were taken from a colony maintained by mating Tc1 females to F1 (129S8×C57BL/6J) males at the National Institute for Medical Research from the Medical Research Center (London, UK) (O’Doherty et al., 2005). Male wildtype (WT) and Tc1 littermates aged, 2.5–3.5 months were used in this study. Animals were housed in plastic cages in groups of 5–7 individuals in the animal facility of the University of Montpellier (CECEMA). They had free access to food and water, except during behavioural experiments, and they were kept in a regulated environment (23±1°C, 40–60% humidity) under a 12-hour light/dark cycle (light on at 08:00), to which they were habituated during at least two weeks before starting behavioural experiments. Experiments were carried out between 09:00 and 17:00, in an experimental room within the animal facility. Mice were habituated 30 min before each experiment. All animal procedures were conducted in strict adherence of European Union Directive of 22 September 2010 (2010/63/UE).

### DNA extraction and genotyping

DNA was extracted from tail tip (approximately 3 mm) or ear biopsy from all samples analysed by either the hot sodium hydroxide and tris (HOTSHOT) method (Truett et al., 2000) or the proteinase K method. For the proteinase K method, tissue is lysed overnight using proteinase K digestion in nuclei lysis buffer (Promega, Madison, Wisconsin, USA), plus 0.12 M ethylenediaminetetracetic acid at 55°C. Proteins are precipitated from the resultant lysate by addition of protein precipitation solution (Promega). DNA is then precipitated with isopropanol and resuspended in DNase free water. Tc1 mice were genotyped using polymerase chain reaction (PCR) (Tc1-specific primers forward: 5′-GGTTTGAGGGAACACAAAGCTTAACTCCCA-3′; reverse: 5′-ACAGAGCTACAGCCTCTGACACTATGAACT-3′; control primers forward: 5′- TTACGTCCATCGTGGACAGCAT-3′; reverse: 5′-TGGGCTGGGTGTTAGTCTTAT-3′). Presence of human *DYRK1A* in Tc1 mice was checked by PCR of genomic DNA using primers specific to human *DYRK1A* sequence (forward 1: 5′- ATCCTCCTCGGGAAGAAGCC-3′, reverse 1: 5′-GTGCATTGTCCTTGCGAATC-3′; forward, 2: 5′-AGCCGAGGAGAGACTGAGCAG-3′; reverse, 2: 5′-AGCCGGCCCCATTTTCTTAAC-3′).

### Aβ_25-35_ administration procedures

**Figure 1.**
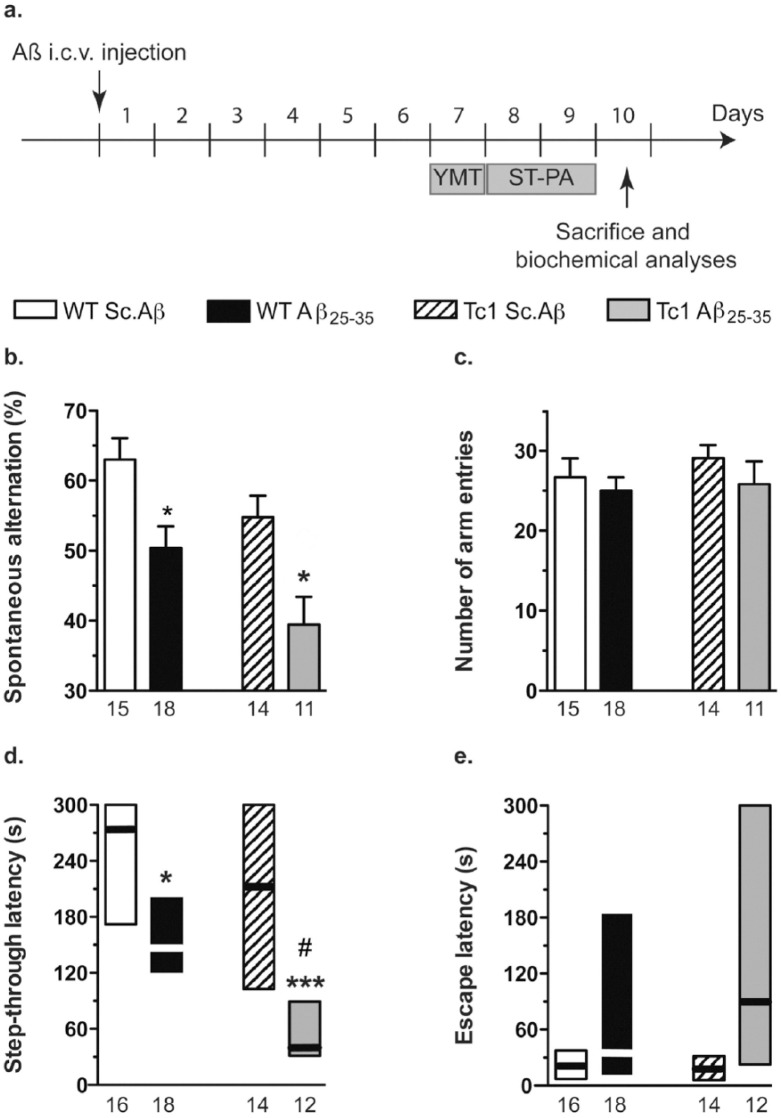
Amyloid-β [25-35] (Aβ_25-35_)-induced memory impairments in Tc1 mice. (a) wildtype (WT) and Tc1 mice were administered intracerebroventricular (i.c.v.) injection with scrambled peptide (Sc.Aβ) or Aβ_25-35_ peptide (9 nmol) at day 0. At day 7, they were tested in the Y-maze: alternation performance (b) and number of arm entries (c). At days 8–9, mice were tested for passive avoidance response: step-through latency (d) and escape latency (e). The number of animals per group is indicated below the columns. Two-way analysis of variance (ANOVA): *F*_(1,54)_=8.42, *p<*0.01 for the genotype, *F*_(1,54)_=17.9, *p<*0.0001 for the treatment, *F*_(1,54)_=0.167, *p*>0.05 for the interaction, in (b); *F*_(1,54)_=1.34, *p>*0.05 for the treatment, *F*_(1,54)_=0.574 for the genotype, *F*_(1,54)_=0.138, *p*>0.05 for the interaction, in (c); *H*=31.2, *p<*0.0001 in (d); *H*=8.72, *p<*0.05 in (e). *p<0.05; **p<0.01; ***p<0.001 vs same genotype Sc.Aβ-treated mice; #*p*<0.05 vs same treatment WT mice; Bonferroni’s test in (b), Dunn’s test in (d) and (e).

The Aβ_25-35_ and Sc.Aβ peptides were purchased from Genepep (Saint-Jean-de-Védas, France), and were solubilised in sterile distilled water at a concentration of 3 mg/mL and stored at −20°C until use. Before injection, peptides were incubated at 37°C for four days, allowing Aβ_25-35_, but not Sc.Aβ, to form oligomers (Zussy et al., 2011, 2013). Mice were injected once, i.c.v. (9 nmol), and under gaseous anaesthesia with isoflurane, as previously described (Maurice et al., 1996; Meunier et al., 2006; Villard et al., 2009, 2011). The experimenter was trained in the hand-free method described by Haley and McCormick (1957), used a Hamilton microsyringe with a 2.5 mm needle (26 gauge) and injected a final volume of 3 µL per mouse, at 3 µL/min. The injection coordinates were −0.4 mm with respect to bregma, ±1.00 mm to the right from the central, and 2.50 mm in depth, corresponding to the top of the lateral ventricle (Paxinos and Franklin, 2004). The injection site was checked in a control group of mice, injected with Indian ink with a visual check of the dissected brains. WT and Tc1 mice were attributed randomly and equally to the four experimental groups. Animals were tested for spontaneous alternation seven days after peptide injection and for passive avoidance response at days 8–9. The animals were assessed randomly to each behavioural test and the experimenter was blind to the genetic and treatment status of animals. They were then sacrificed on day 10 and cortex and hippocampus were removed from skull for biochemical analyses (Figure 1(a)).

### Spontaneous alternation performances

The Y-maze is made of grey polyvinylchloride and placed in a dimmed light room (150 Lux). Each arm is 40 cm long, 13 cm high, 3 cm wide at the bottom, 10 cm wide at the top, and converging at an equal angle. Each mouse, naive to the apparatus, was placed at the end of one arm and allowed to move freely throughout the maze during a single eight-minute session. The series of arm entries, including possible returns into the same arm, was recorded visually. An alternation was defined as entries into all three arms on consecutive choices. The number of the total possible alternations was therefore the total number of arm entries minus two and the percentage of alternation was calculated as: (actual alternations/total possible alternations)×100. Animals performing less than eight arm entries in eight minutes were discarded from the calculation. In this study, two mice were discarded accordingly, one Sc.Aβ-treated and one Aβ_25-35_-treated Tc1 mice, corresponding to 3.4% attrition.

### Step-through type passive avoidance test

The apparatus consisted of two identically sized compartments (15×20×15 cm high), one illuminated with white polyvinylchloride walls and, one darkened with black polyvinylchloride walls and a grid floor. A guillotine door separated each compartment. A 60 W lamp positioned 40 cm above the apparatus lit the white compartment during the experimental period. Scrambled foot shocks (0.3 mA for three seconds) were delivered to the grid floor using a shock generator scrambler (Lafayette Instruments, Lafayette, Massachusetts, USA). The guillotine door was initially closed during the training session. Each mouse was placed into the white compartment. After five seconds, the door was raised. When the mouse entered the darkened compartment and placed all its paws on the grid floor, the door was gently closed and the scrambled foot shock was delivered for three seconds. The step-through latency, i.e. the latency spent to enter the dark compartment, and the level of sensitivity to the shock were recorded. The latter was evaluated as: 0=no sign; 1=flinching reactions;, 2=flinching and vocalization reactions. None of the treatments used in the present study affected the step-through latency or shock sensitivity during training sessions (not shown). The retention test was carried out 24 h after training. Each mouse was placed again into the white compartment. After five seconds, the door was raised. The step-through latency and escape latency, i.e. the latency spent to re-exit from the dark compartment, were recorded up to 300 s. Animals that showed all latencies during the training and retention session lower than 10 s were considered as failing to respond to the procedure and were discarded from the calculations. In this study, no animal was discarded accordingly.

### Animal sacrifice and tissue collection

Animals were sacrificed, 24 h after the retention test of the passive avoidance. Brains were rapidly removed from the skulls. Hemibrains were then separated and olfactory bulbs and cerebellum were removed. Each hemibrain was dissected out to separate hippocampus and cortex, which were rapidly frozen in liquid nitrogen and stored at −80°C until protein analysis. One hemibrain (cortex and hippocampus) was used for Western blot analyses, whereas the second hemibrain (cortex and hippocampus) was taken to determine the reactive oxygen species (ROS) content and to perform enzyme-linked immunosorbent assays (ELISA). For each biochemical analysis, the samples were examined randomly.

### Measurement of ROS

The accumulation of ROS was determined by analysing dichlorofluorescein (DCF) fluorescence. For determination of ROS accumulation, DCF-diacetate, 0.5 µM (Sigma-Aldrich, Saint Louis, Missouri, USA) was applied to the protein extract of cortex and hippocampus. The free DCF-diacetate was readily converted into DCF, which is able to interact with peroxides (primarily H_2_O_2_) to form the highly fluorescent DCF. DCF fluorescence was quantified (excitation at 485 nm, emission at 530 nm) using a Fluoroskan Ascent spectrofluorimeter (Thermo Scientific, Waltham, USA), normalised for protein concentration.

### Synaptic marker measures

One hippocampus per mouse was homogenised in cold phosphate-buffered saline. Synaptic marker content – Arc, Egr1, PSD-95 and synaptophysin – was analysed using an ELISA kit (USCN Life Science, Euromedex, Souffelweyersheim, France). Levels of synaptic markers were expressed as ng/mg hippocampus wet weight.

### Western blotting

For determination of protein levels, the cortex and half-hippocampus were homogenised in a lysis buffer (125 mM Tris-HCl pH 6.8, containing 4% sodium dodecyl sulfate, 20% glycerol) including a protease and phosphatase inhibitors cocktail (Roche Diagnostics, Germany). Homogenates were heated at 70°C for 10 min and centrifuged at 16,000 g for 30 min at 4°C. Protein concentration was determined using the Pierce BCA assay (Pierce Biotechnology, Rockford, USA) according to the manufacturer’s instructions. Proteins, 20–40 µg per lane, were resolved on a 12% sodium dodecyl sulfate-polyacrylamide gel. Proteins were then transferred electrophoretically onto a polyvinylidene difluoride membrane (GE Healthcare, France). After one hour blocking in 5% non-fat dry milk in a 20 mM Tris-buffered saline, pH 7.5, buffer containing 0.1% Tween-20, membranes were incubated overnight at 4°C with the primary antibodies (Table 1). After brief washes, membranes were incubated for one hour at room temperature with corresponding secondary antibody (Table 1). The immunoreactive bands were visualised with the enhanced chemiluminescence reagent (Pierce Biotechnology) and quantified using a Licor Odyssey Fc quantitative fluorescence imaging system (LI-COR ScienceTec, France) at the ‘qPHD UM2/Montpellier GenomiX’ facility. Results were corrected with the corresponding β-tubulin (β-tub) level and expressed as percentage of control group data. Each protein level was determined as mean of triplicate determination for each animal and values represented are the mean of the number of animals indicated in the figure legends.

**Table 1.** Antibodies used in the study.

Protein	MW	Antibody	Dilution	Reference	Supplier
*Primary antibodies*:					
DYRK1A	90 KDa	Rabbit anti-DYRK1A	1:2000	2771	Cell Signaling
pAkt	70 KDa	Rabbit anti-P(S473)-Akt	1:2000	9271	Cell Signaling
Akt	70 KDa	Rabbit anti-Akt	1:2000	9272	Cell Signaling
pGSK-3β	46 KDa	Mouse anti-P(Tyr216)-GSK-3β	1:2000	612313	BD Biosciences
pGSK-3β	46 KDa	Rabbit anti-P(Ser9)-GSK-3β	1:2000	9336	Cell Signaling
GSK-3β	46 KDa	Rabbit anti-GSK-3β	1:2000	sc-9166	Santa Cruz
Bax	20 KDa	Rabbit anti-Bax	1:2000	2772	Cell Signaling
Bim	23 KDa	Rabbit anti-Bim	1:1000	B7929	Sigma-Aldrich
Bcl-XL	28 KDa	Rabbit anti-Bcl-XL	1:2000	PA1-14066	Pierce
Bcl-W	18 KDa	Rabbit anti-Bcl-W	1:1000	MA5-15076	Pierce
βTub	49 KDa	Mouse monoclonal anti-β-Tubulin	1:5000	T4026	Sigma-Aldrich
*Secondary antibodies*:					
IgG	Goat anti-rabbit IgG peroxidase conjugate		1:2000	A6154	Sigma-Aldrich
IgG	Goat anti-mouse IgG peroxidase conjugate		1:2000/1:5000	A4416	Sigma-Aldrich

β-tub: β-tubulin; Akt, protein kinase B; DYRK1A: dual specificity, tyrosine phosphorylation regulated kinase 1A; GSF-3β: glycogen synthase kinase-3β: IgG: immunoglobulin G; MW: molecular weight.

### Statistical analyses

Biochemical and behavioral data were expressed as mean±standard error of the mean (SEM). The exact sample size (*n*) for each experimental group is specified for all experiments in each figure. Group sizes were determined empirically as the minimum size to allow pertinent ANOVAs, i.e. *n*=6–12 for biochemical ex vivo analyses and *n*=12–18 for behavioural analyses. In biochemical data, aberrant values, i.e. << mean–3×SEM or >> mean+3×SEM, were discarded from the calculations. This accounted for less than 2% of the data. Animals were not randomised a priori, but the experimenter was blinded to the treatment and genotype during experiments. These were analysed using two-way analysis of variance (ANOVA) (*F* values), with genotype and treatment as independent factors, followed by Bonferroni’s test for planned multiple comparisons. Homogeneity of the variances at the interaction was checked systematically using Bartlett’s test. Passive avoidance latencies are non-parametric data, since upper cut-off times were set, and were expressed as median and interquartile range. They were analysed using a Kruskal–Wallis nonparametric ANOVA (*H* values), followed by Dunn’s multiple comparison test. For reading clarity, all statistical values are detailed in the figure legends. The level of statistical significance considered was *p*<0.05.

## Results

### Aβ_25-35_-induced memory impairments are increased in Tc1 mice

Spatial working memory was assessed using spontaneous alternation in the Y-maze seven days after i.c.v. injection (Figure 1(a)–(c)). Aβ_25-35_ treatment induced a significant decrease of alternation performance in WT mice. In Tc1 mice, results were generally lower than in WT mice. Scrambled Aβ_25-35_ peptide (Sc.Aβ)-treated Tc1 mice tended to show a decreased alternation performance as compared with Sc.Aβ-treated WT mice, but the difference was non-significant (*p*>0.05). Aβ_25-35_ also induced an alternation deficit in Tc1 mice (Figure 1(b)). Notably, the alternation percentage observed was markedly below 50% suggesting that, in Tc1 mice, Aβ_25-35_ induced major cognitive deficits involving both working memory blockade and perseverative deficits. In parallel, the genotype or treatment did not affect the measure of numbers of arm entries (Figure 1(c)).

Non-spatial long-term memory was assessed using the passive avoidance procedure on days 8–9 after i.c.v. injection (Figure 1(a), (d), (e)). Neither the treatment nor the genotype affected the step-through latency or sensitivity to the shocks during the training session (data not shown). During the retention session, Aβ_25-35_ treatment provoked a significant decrease in step-through latency (Figure 1(d)). No difference was measured between Sc.Aβ-treated WT and Tc1 mice, but the Aβ_25-35_-induced deficit was significantly increased in Tc1 mice in terms of step-through latency, compared to Sc.Aβ-treated animals (Figure 1(d)). Kruskal-Wallis analysis of escape latency showed a significant overall difference between the four groups but group comparison only led to a non-significant increase in escape latency in both WT and Tc1 Aβ_25-35_-treated animals (*p*>0.05; Figure 1(e)).

### Increased expression of DYRK1A in hippocampus and frontal cortex of Tc1 mice, injected with Aβ_25-35_

**Figure 2.**
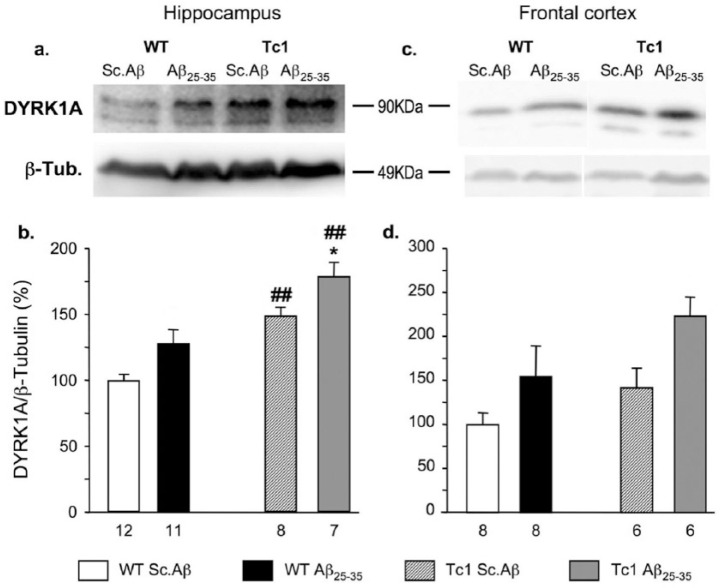
Expression of dual specificity, tyrosine phosphorylation regulated kinase 1A (DYRK1A) protein in the hippocampus ((a) and (b)) and cortex ((c) and (d)) of Tc1 mice injected with amyloid-β [25-35] (Aβ_25-35_) peptide. Wildtype (WT) or Tc1 mice were administered intracerebroventricular (i.c.v.) injections with scrambled peptide (Sc.Aβ) or Aβ_25-35_ peptide (9 nmol) and sacrificed 10 days after injection. The levels of DYRK1A proteins were assessed by Western blotting. Typical blots are shown in ((a) and (c)). The number of animals per group is indicated below the columns in ((b) and (d)). Two-way analysis of variance (ANOVA): *F*_(1,34)_=32.7, *p<*0.0001 for the genotype, *F*_(1,34)_=11.0, *p<*0.01 for the treatment, *F*_(1,34)_=0.011, *p*>0.05 for the interaction in (b); *F*_(1,24)_=7.17, *p<*0.05 for the genotype, *F*_(1,24)_=4.68, *p<*0.05 for the treatment, *F*_(1,24)_=0.278, *p*>0.05 for the interaction in (d). *p<0.05 vs same genotype Sc.Aβ-treated mice; ##*p<*0.01 vs same treatment WT mice; Bonferroni’s test.

Protein level of DYRK1A was assessed in the hippocampus and frontal cortex of WT and Tc1 mice treated with Sc.Aβ or Aβ_25-35_ using Western blot analysis 10 days after i.c.v. injection. Two-way ANOVA showed that treatment had an impact on DYRK1A expression in the two structures and a genotype effect was observed in the hippocampus and in the cortex (Figure 2). DYRK1A level was significantly increased in Tc1 mice compared to WT in the hippocampus (Figure 2(a) and (b)) and non-significantly in the cortex (Figure 2(c) and (d)). The Aβ_25-35_ treatment did not induce a significant increase of DYRK1A expression in WT and Tc1 mouse hippocampus (Figure 2(a) and (b)) or in the frontal cortex of Tc1 mice (Figure 2(c) and (d)).

### Oxidative stress is increased in the cortex of Tc1 mice

**Figure 3.**
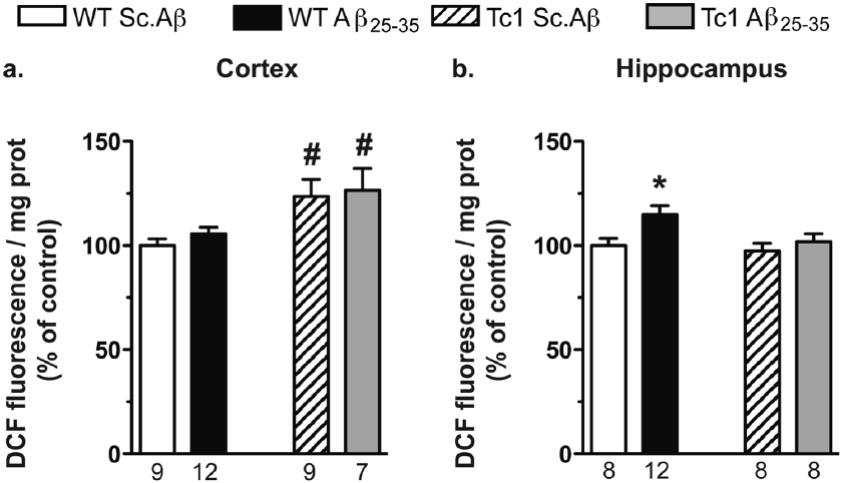
Tc1 mice exhibited increased oxidative stress in the cortex. Oxidative stress was assessed 10 days after intracerebroventricular (i.c.v.) amyloid-β [25-35] (Aβ_25-35_) injection by measuring reactive oxygen species (ROS) accumulation using dichlorofluorescein (DCF) fluorescence in mouse cortex (a) and hippocampus (b). The number of animals per group is indicated below the columns. Two-way analysis of variance (ANOVA): *F*_(1,33)_=12.5, *p<*0.01 for the genotype, *F*_(1,33)_=0.467, *p*>0.05 for the treatment, *F*_(1,33)_=0.049; *p*>0.05 for the interaction in (a); *F*_(1,32)_=3.62, *p>*0.05 for the genotype, *F*_(1,32)_=5.51, *p<*0.05 for the treatment, *F*_(1,32)_=1.61, *p>*0.05 for the interaction in (b); *p<0.05 vs same genotype scrambled peptide (Sc.Aβ)-treated mice; #*p<*0.05 vs same treatment wildtype (WT) mice; Bonferroni’s test.

To measure oxidative stress, an indirect quantification of ROS accumulation using the measure of DCF fluorescence was performed in cortex and hippocampus 10 days after Aβ_25-35_ injection. Two-way ANOVAs revealed a group effect of genotype in the cortex and treatment in the hippocampus (Figure 3). Aβ_25-35_ failed to alter ROS level in WT mice in the cortex (Figure 3(a)), but increased it significantly in the hippocampus (Figure 3(b)). Sc.Aβ-treated Tc1 mice showed an increased level of DCF fluorescence in cortex (Figure 3(a)), but not in the hippocampus (Figure 3(b)). Aβ_25-35_ did not increase DCF fluorescence in Tc1 mice in both structures (Figure 3(a) and (b)).

### Synaptic integrity in Tc1 mice, injected with Aβ_25-35_

**Figure 4.**
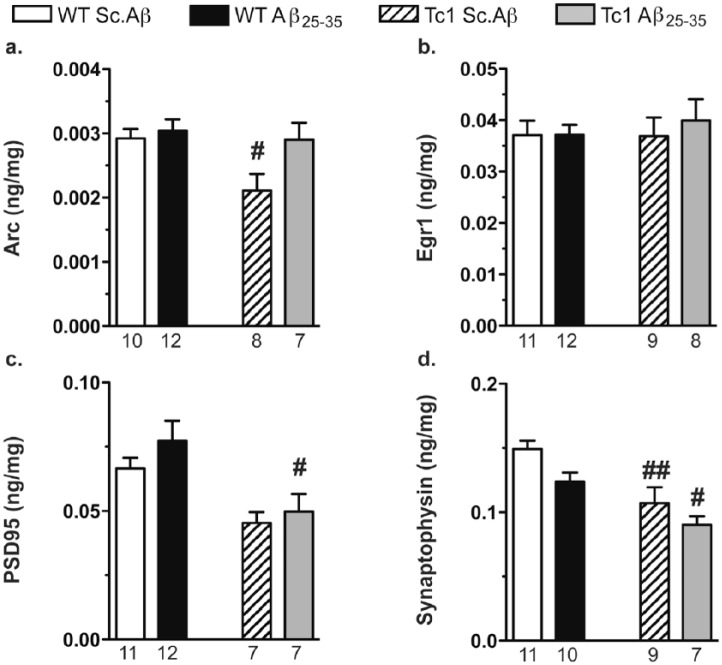
Expression of synaptic markers in the hippocampus of Tc1 mice injected with amyloid-β [25-35] (Aβ_25-35_) peptide. Wildtype (WT) or Tc1 mice were administered intracerebroventricular (i.c.v.) injections with scrambled peptide (Sc.Aβ) or Aβ_25-35_ peptide (9 nmol) and sacrificed 10 days after injection. The levels of synaptic markers were assessed in the half-hippocampus by enzyme-linked immunosorbent assay and reported to the level of proteins (mg): Arc (a), Egr1 (b), PSD95 (c) and synaptophysin (d). The number of animals per group is indicated below the columns. Two-way analysis of variance (ANOVA): *F*_(1,33)_=5.11, *p<*0.05 for the genotype, *F*_(1,33)_=4.69, *p<*0.05 for the treatment, *F*_(1,33)_=2.54, *p>*0.05 for the interaction in (a); *F*_(1,36)_=0.173, *p>*0.05 for the genotype, *F*_(1,36)_=0.261, *p>*0.05 for the treatment, *F*_(1,36)_=0.232, *p>*0.05 for the interaction in (b); *F*_(1,33)_=13.6, *p<*0.001 for the genotype, *F*_(1,33)_=1.33, *p>*0.05 for the treatment, *F*_(1,33)_=0.230, *p>*0.05 for the interaction in (c); *F*_(1,33)_=19.4, *p<*0.0001 for the genotype, *F*_(1,33)_=6.05, *p<*0.05 for the treatment, *F*_(1,33)_=0.253, *p>*0.05 for the interaction in (d); #*p<*0.05, ##*p<*0.01 vs same treatment WT mice; Bonferroni’s test.

Synaptic integrity was assessed in the mouse hippocampus by determining the level of several synaptic markers, 10 days after Aβ_25-35_ injection. Two-way ANOVAs showed a genotype effect for Arc, PSD95 and Synaptophysin and a treatment effect for Arc and Synaptophysin (Figure 4). The levels of Arc, Egr1 and PSD95 were not affected by Aβ_25-35_ injection in WT mice, as compared with Sc.Aβ-treated WT animals (Figure 4(a)–(c)). Tc1 mice showed a decreased Arc level (Figure 4(a)), no change in Egr1 (Figure 4(b)) and a trend to decreased PSD95 level (Figure 4(c)). The Aβ_25-35_-treatment increased Arc level (Figure 4(a)) and decreased PSD95 level in Tc1 mice, as compared to Aβ_25-35_-treated WT mice (Figure 4(c)). Synaptophysin level was lowered by ~17% in WT mice treated with Aβ_25-35_ (*p*>0.05, Figure 4(d)) and very significantly decreased in Tc1 compared to WT animals. The Aβ_25-35_-treatment in Tc1 mice decreased synaptophysin level by −16%, as compared with Sc.Aβ-treated animals (Figure 4(d)).

### Aβ_25-35_ effects on GSK-3β phosphorylation in Tc1 mice

**Figure 5.**
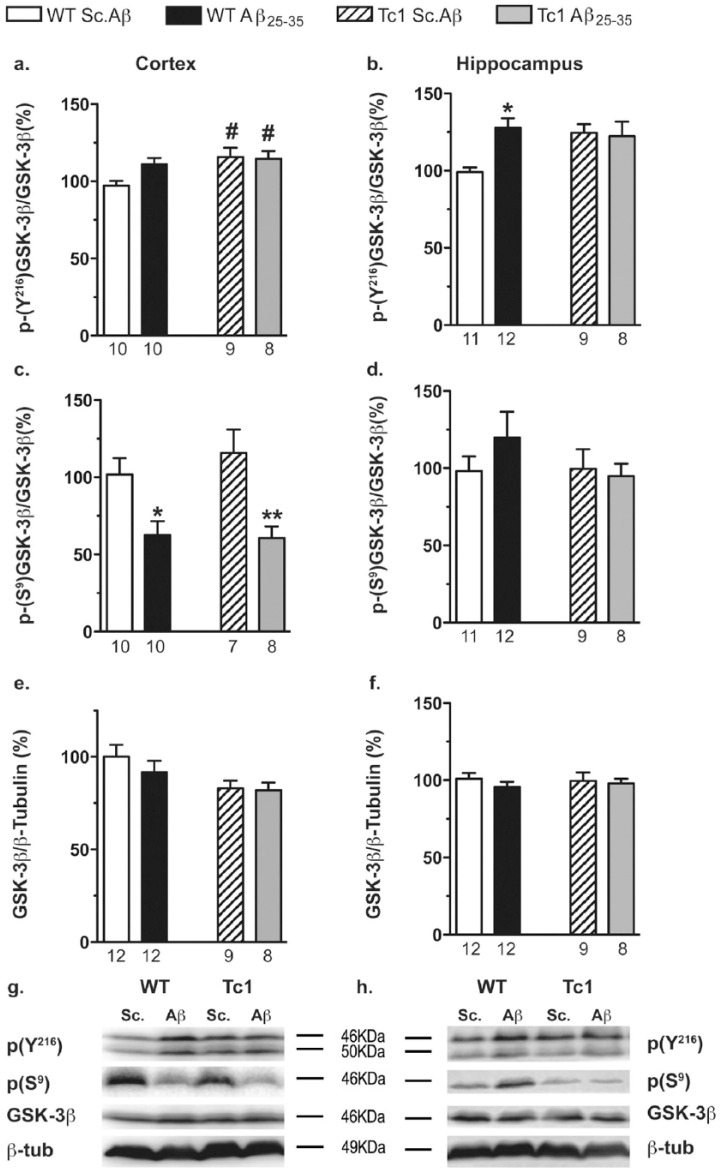
Phosphorylation of glycogen synthase kinase-3β (GSK-3β) in the cortex and hippocampus of Tc1 mice injected with amyloid-β [25-35] (Aβ_25-35_) peptide. Wildtype (WT) or Tc1 mice were administered intracerebroventricular (i.c.v.) injections with scrambled peptide (Sc.Aβ) or Aβ_25-35_ peptide (9 nmol) and sacrificed 10 days after injection. The levels of GSK-3β phosphorylated at tyrosine, 216 ((a) and (b)) and serine-9 ((c) and (d)) total and GSK-3β ((e) and (f)) were assessed by Western blot in the cortical ((a), (c), (e)) and hippocampal ((b), (d), (f)) protein lysates. Typical blots are shown for the cortex (g) and the hippocampus (h). The number of animals per group is indicated below the columns. Two-way analysis of variance (ANOVA): *F*_(1,33)_=6.04, *p<*0.05 for the genotype, *F*_(1,33)_=1.88, *p>*0.05 for the treatment, *F*_(1,33)_=2.69, *p>*0.05 for the interaction in (a); *F*_(1,36)_=1.61, *p>*0.05 for the genotype, *F*_(1,36)_=4.61, *p<*0.05 for the treatment, *F*_(1,36)_=2.50, *p>*0.05 for the interaction in (b); *F*_(1,30)_=20.0, *p<*0.0001 for the treatment, *F*_(1,30)_=0.323, *p>*0.05 for the genotype, *F*_(1,30)_=0.566, *p>*0.05 for the interaction in (c); *F*_(1,36)_=0.800, *p>*0.05 for the genotype *F*_(1,36)_=0.414, *p>*0.05 for the treatment, *F*_(1,36)_=1.01, *p>*0.05 for the interaction in (d); *F*_(1,37)_=5.12, *p<*0.05 for the genotype, *F*_(1,37)_=0.622, *p>*0.05 for the treatment, *F*_(1,37)_=0.374, *p>*0.05 for the interaction in (e); *F*_(1,37)_=0.011, *p>*0.05 for the genotype, *F*_(1,37)_=0.679, *p>*0.05 for the treatment, *F*_(1,37)_=0.223, *p>*0.05 for the interaction in (f); *p<0.05; **p<0.01 vs same genotype Sc.Aβ-treated mice; #*p<*0.05 vs same treatment WT mice; Bonferroni’s test.

To assess GSK-3β activity, we determined the amount and phosphorylation status of GSK-3β in cortex and hippocampus of WT and Tc1 mice injected with Sc.Aβ or Aβ_25-35_. GSK-3β is constitutively active and its activity is correlated with the phosphorylation of GSK-3β at Tyr^216^ (Hughes et al., 1993; Medina and Wandosell, 2011). Phosphorylation at other sites is known to modulate GSK-3β activity. Phosphorylation of Ser^9^ significantly inhibits GSK-3β activity by blocking the substrate access to the catalytic site (Cohen and Frame, 2001; Medina and Wandosell, 2011; Sutherland et al., 1993). In the cortex, two-way ANOVAs revealed a genotype effect for GSK-3β phosphorylation at Tyr^216^ (Figure 5(a)) and for GSK-3β levels (Figure 5(e)) and a treatment effect for GSK-3β phosphorylation at Ser^9^ (Figure 5(c)). In the hippocampus, a treatment effect was observed for GSK-3β phosphorylation at Tyr^216^ (Figure 5(b)) and no effect for both GSK-3β phosphorylation at Ser^9^ (Figure 5(d)) and GSK-3β levels (Figure 5(f)). Tc1 mice showed a significant increase in GSK-3β phosphorylation at Tyr^216^ (Figure 5(a)) and a trend to decrease level of GSK-3β (Figure 5(e)), which is coherent with a constitutive activation of GSK-3β in the cortex of Tc1 mice. Aβ_25-35_ decreased GSK-3β phosphorylation at Ser^9^ (Figure 5(c)) in WT as well as in Tc1 mice, showing that the peptide also activated GSK-3β in the cortex, similarly in both genotypes, through the Ser^9^ site. In the hippocampus of WT mice, the peptide significantly increased phosphorylation of GSK-3β at Tyr^216^ (Figure 5(b)). However, no change was seen in Tc1 mice, as compared with WT and after injection of the peptide, suggesting that Aβ_25-35_ failed to activate GSK-3β.

**Figure 6.**
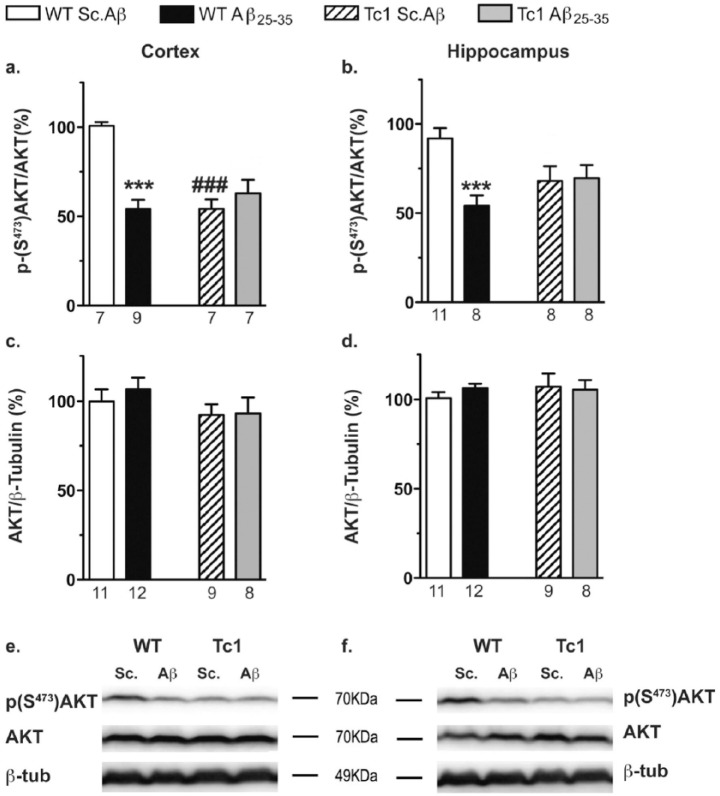
Phosphorylation of protein kinase B (AKT) at serine 473 in the cortex and hippocampus of Tc1 mice injected with amyloid-β [25-35] (Aβ_25-35_) peptide. Wildtype (WT) or Tc1 mice were administered intracerebroventricular (i.c.v.) injections with scrambled peptide (Sc.Aβ) or Aβ_25-35_ peptide (9 nmol) and sacrificed 10 days after injection. The abundance of AKT phosphorylated at serine 473 ((a) and (b)) and total AKT ((c) and (d)) was investigated by Western blot of total cortical ((a) and (c)) and hippocampal ((b) and (d)) protein lysates. Phospho-AKT signal was normalised to total AKT signal in each group. Typical blots are shown for the cortex (e) and the hippocampus (f). The number of animals per group is indicated below the columns. Two-way analysis of variance (ANOVA): *F*_(1,26)_=12.4, *p<*0.01 for the genotype, *F*_(1,26)_=12.4, *p<*0.01 for the treatment, *F*_(1,26)_=26.4, *p<*0.0001 for the interaction in (a); *F*_(1,31)_=0.385, *p>*0.05 for the genotype, *F*_(1,31)_=7.06, *p*<0.05 for the treatment, *F*_(1,31)_=8.29, *p<*0.01 for the interaction in (b); *F*_(1,37)_=2.22, *p>*0.05 for the genotype, *F*_(1,37)_=0.285, *p*>0.05 for the treatment, *F*_(1,37)_=0.172, *p*>0.05 for the interaction in (c); *F*_(1,36)_=0.371, *p>*0.05 for the genotype, *F*_(1,36)_=0.184, *p>*0.05 for the treatment, *F*_(1,36)_=0.604, *p>*0.05 for the interaction in (d). ***p<0.001 vs same genotype Sc.Aβ-treated mice; ###*p<*0.001 vs same treatment WT mice; Bonferroni’s test.

AKT is one of the main kinases phosphorylating GSK-3β on its inhibitory residue, Ser^9^ (Cross et al., 1995). We therefore measured the level of AKT phosphorylation at Ser^473^ (Figure 6). Two-way ANOVAs showed an effect of genotype for AKT phosphorylation at Ser^473^ in the cortex (Figure 6(a)), effects for treatment and interaction in both structures (Figure 6(a) and (b)) and no effect for AKT level (Figure 6(c) and (d)). Indeed, Aβ_25-35_ decreased AKT phosphorylation at Ser^473^ highly significantly both in cortex and hippocampus of WT mice (Figure 6(a) and (b)). Tc1 mice presented decreased levels of AKT phosphorylation at Ser^47^, significant in the cortex but Aβ_25-35_ injection had no additional effect on phosphorylation level of AKT in both structures in Tc1 mice (Figure 6(a) and (b)). Thus, the Aβ_25-35_–induced decreased phosphorylation of GSK-3β at Ser^9^ in Tc1 mice could not be attributable to a modification of AKT activity.

### Apoptotic markers in Tc1 mice, injected with Aβ_25-35_

**Figure 7.**
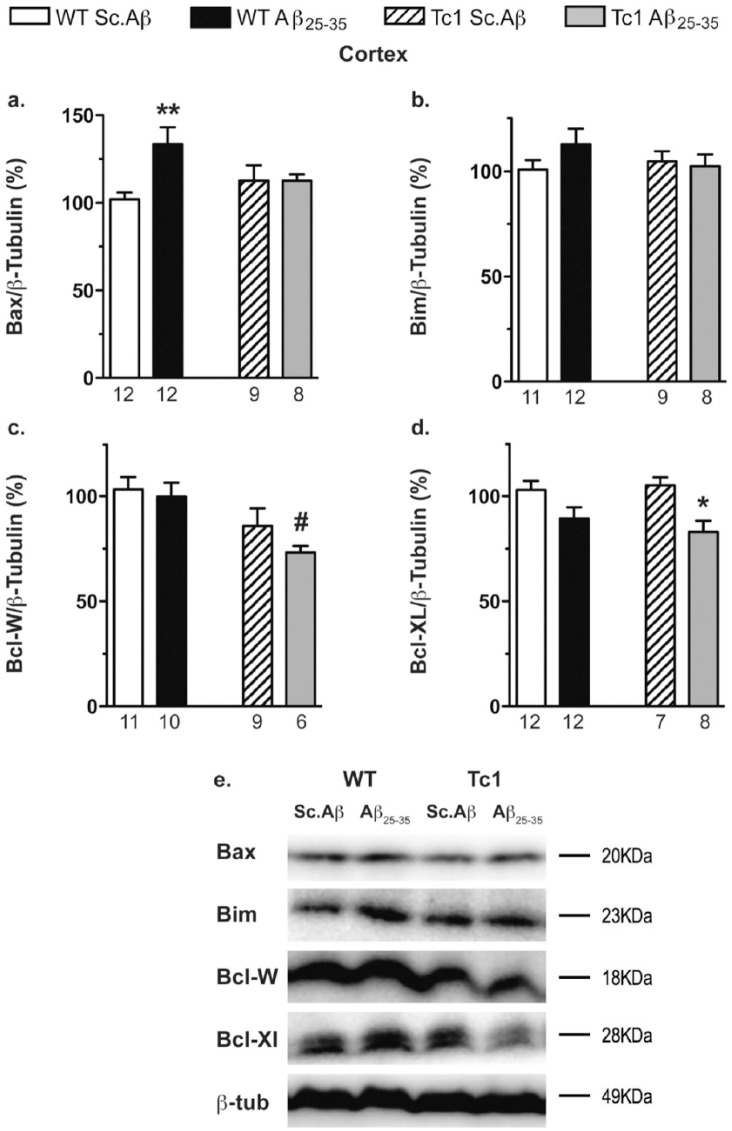
Analyses of apoptotic pathway in the cortex of Tc1 mice injected with amyloid-β [25-35] (Aβ_25-35_) peptide. Wildtype (WT) or Tc1 mice were administered intracerebroventricular (i.c.v.) injections with scrambled peptide (Sc.Aβ) or Aβ_25-35_ peptide (9 nmol) and sacrificed 10 days after injection. The levels of pro-apoptotic proteins, Bax (a) and Bim (b), and anti-apoptotic proteins, Bcl-W (c) and Bcl-XL (d), were assessed by Western blot in the cortical protein lysates. Typical blots are shown in (e). The number of animals per group is indicated below the columns. Two-way analysis of variance (ANOVA): *F*_(1,26)_=0.477, *p>*0.05 for the genotype, *F*_(1,26)_=4.43, *p<*0.05 for the treatment, *F*_(1,37)_=4.54, *p<*0.05 for the interaction in (a); *F*_(1,36)_=0.302, *p*>0.05 for the genotype, *F*_(1,36)_=0.649, *p*>0.05 for the treatment, *F*_(1,36)_=1.40, *p*>0.05 for the interaction in (b); *F*_(1,32)_=10.2, *p<*0.01 for the genotype, *F*_(1,32)_=1.33, *p>*0.05 for the treatment, *F*_(1,32)_=0.432, *p>*0.05 for the interaction in (c); *F*_(1,35)_=12.3, *p<*0.01 for the treatment, *F*_(1,35)_=0.181, *p>*0.05 for the genotype, *F*_(1,35)_=0.719, *p>*0.05 for the interaction in (d). *p<0.05; **p<0.01 vs same genotype Sc.Aβ-treated mice; #*p<*0.05 vs same treatment WT mice; Bonferroni’s test.

In the cortex, Aβ_25-35_ induced an increase of the proapoptotic protein Bax in WT mice (Figure 7(a) and (e)), but without affecting Bim (Figure 7(b) and (e)), Bcl-W (Figure 7(c) and (e)) or Bcl-XL levels (Figure 7(d) and (e)). In Sc.Aβ-treated Tc1 mice, the two-way ANOVA showed a genotype effect only for Bcl-W (Figure 7(c) and (e)) but no change for other pro- or anti-apoptotic proteins compared to Sc.Aβ-treated WT mice (Figure 7(a), (b), (d)). The Aβ_25-35_-treatment induced in Tc1 mice significant decreases of anti-apoptotic proteins, Bcl-W (Figure 7(c)) and Bcl-XL (Figure 7(d)).

**Figure 8.**
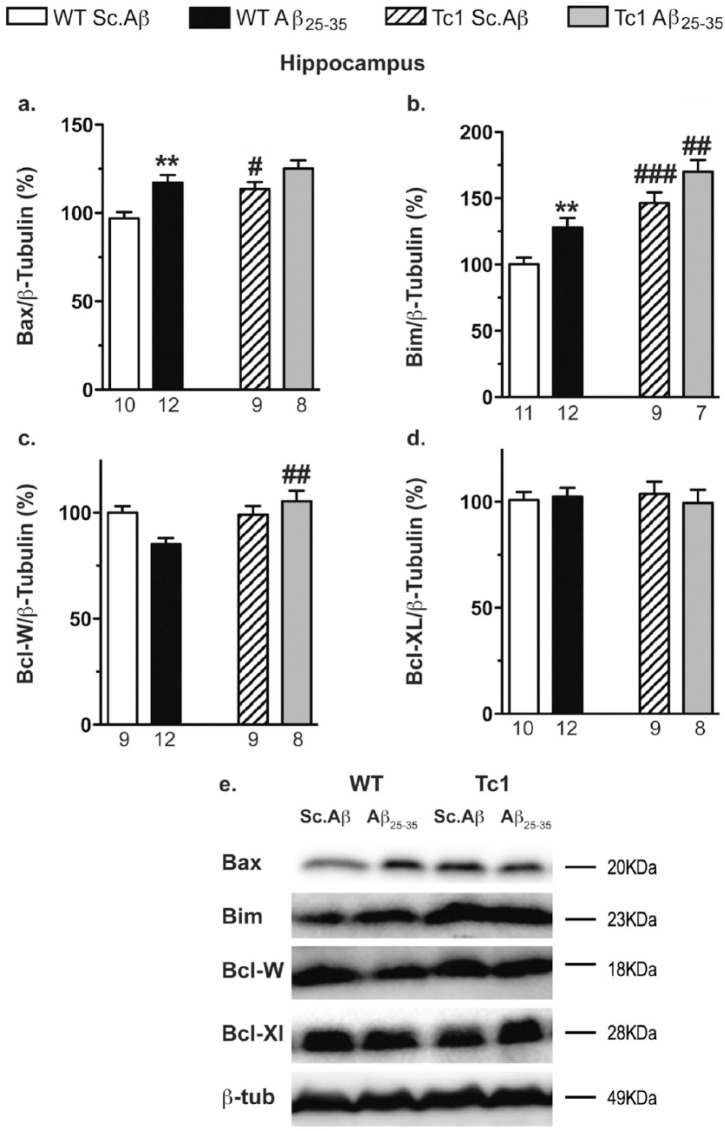
Analyses of apoptotic pathway in the hippocampus of Tc1 mice injected with amyloid-β [25-35] (Aβ_25-35_) peptide. Wildtype (WT) or Tc1 mice were administered intracerebroventricular (i.c.v.) injections with scrambled peptide (Sc.Aβ) or Aβ_25-35_ peptide (9 nmol) and sacrificed 10 days after injection. The levels of pro-apoptotic proteins, Bax (a) and Bim (b), and anti-apoptotic proteins, Bcl-W (c) and Bcl-XL (d), were assessed by Western blot in the hippocampal protein lysates. Typical blots are shown in (e). The number of animals per group is indicated below the columns. Two-way analysis of variance (ANOVA): *F*_(1,35)_=8.37, *p*<0.01 for the genotype, *F*_(1,35)_=14.1, *p*<0.001 for the treatment, *F*_(1,35)_=1.03, *p*>0.05 for the interaction in (a); *F*_(1,36)_=34.8, *p*<0.0001 for the genotype, *F*_(1,36)_=10.1, *p*<0.01 for the treatment, *F*_(1,36)_=0.559, *p>*0.05 for the interaction in (b); *F*_(1,33)_=6.28, *p*<0.05 for the genotype, *F*_(1,33)_=1.22, *p*>0.05 for the treatment, *F*_(1,37)_=7.71, *p*<0.01 for the interaction in (c); *F*_(1,30)_=0.463, *p*>0.05 for the genotype, *F*_(1,30)_=1.47, *p*>0.05 for the treatment, *F*_(1,30)_=1.48, *p*>0.05 for the interaction in (d); *p<0.05; **p<0.01 vs same genotype Sc.Aβ-treated mice; #*p*<0.05; ##*p* 0.01; ###*p*<0.001 vs same treatment WT mice; Bonferroni’s test.

In the hippocampus, Aβ_25-35_ injection significantly increased both pro-apoptotic proteins Bax (Figure 8(a) and (e)) and Bim (Figure 8(b) and (e)) in WT mice and failed to affect the anti-apoptotic markers Bcl-W (Figure 8(c)) and Bcl-XL (Figure 8(d)). A two-way ANOVA showed a genotype effect for Bax, Bim and Bcl-W in Tc1 mice. In particular the pro-apoptotic markers were increased. The Aβ_25-35_ injection resulted in a mild further increase in each marker (Figure 8(a) and (b)). No modification of Bcl-XL level was observed in hippocampus of Tc1 mice, injected or not with Aβ_25-35_ (Figure 8(d) and (e)).

In conclusion, WT and Tc1 mice showed a differential vulnerability to Aβ_25-35_-induced apoptosis among brain structures. The peptide increased pro-apoptotic markers in both structures whereas it decreased anti-apoptotic proteins, in the cortex of Tc1 mice.

## Discussion

The prevalence of AD is increased in people with DS and the hallmarks of AD appear earlier than in the general population, suggesting that triplication of Hsa21 genes may favour the onset of AD pathology. The mechanism underlying this vulnerability is however, not fully understood. Here, we examined whether trisomy of Hsa21 genes altered the toxicity induced by an oligomeric amyloid peptide. We compared the toxicity induced by Aβ_25-35_ oligomers in three-month old WT and Tc1 mice, the unique humanised model for DS trisomic for 75% of Hsa21 (Gribble et al., 2013). The acute model of Aβ toxicity, consisting in an i.c.v. injection of oligomerised Aβ_25-35_, induced rapidly and sustainably an AD-like toxicity and memory impairments (Chavant et al., 2000; Delobette et al., 1997; Kaminsky et al., 2010; Klementiev et al., 2007; Maurice et al., 1996; Meunier et al., 2006; Villard et al., 2009, 2011; Zussy et al., 2011). In particular, numerous aspects of the toxicity (oxidative stress, neuroinflammation, apoptosis, synaptic and cellular losses, amyloid accumulation, Tau hyperphosphorylation and activation of the related kinases) are observed. The model thus allows the screening of protective agents or vulnerability conditions for AD toxicity. The model also has a high predictive value for testing neuroprotectants towards transgenic models. For instance, we previously reported that Neuro-EPO, a low sialic acid form of erythropoietin, attenuated memory deficits and toxicity markers in Aβ_25-35_-injected mice (Maurice et al., 2013) at a similar dose as observed after a two-month treatment in APP_Swe_ mice (Rodríguez Cruz et al., 2017). In this study, we show that Aβ_25-35_ treatment rapidly provokes in WT mice a decline in spatial working and contextual long-term memory and the induction of numerous markers of neurotoxicity: oxidative stress, decrease in hippocampal synaptophysin level, activation of GSK-3β, decreased activity of AKT and increase pro-apoptotic markers in the cortex and hippocampus. A pre-existing neurotoxicity was observed in Tc1 mice, induced by Hsa21 genes, with increased ROS level in the cortex, decreases in hippocampal synaptic markers, increased GSK-3β activity and decreased AKT activity in both structures, and increased Bax and Bim levels in the hippocampus. Furthermore, injection of Aβ_25-35_ resulted however in learning impairment, to a greater extent that was seen in WT mice. Particularly, the Aβ_25-35_-induced a decline in contextual long-term memory is significantly enhanced in Tc1 mice. Moreover, Aβ_25-35_ still decreased hippocampal synaptophysin level, activated GSK-3β and decreased Bcl-W or Bcl-XL levels in the cortex in *Tc1* mice. However, several alterations were not observed in Tc1 mice after Aβ_25-35_ injection, including ROS generation, decrease in synaptophysin level, induction of GSK-3β activity in the hippocampus and reduction of AKT activity in both cortex and hippocampus. These observations suggest that amyloid toxicity develops differently in WT and Tc1 mice.

The strong phenotype of young Sc.Aβ-treated Tc1 mice is unlikely to result from an effect of this scrambled peptide, which is unable to oligomerise and to induce any biological effect when injected i.c.v. in rodents (Brureau et al., 2013; Delobette et al., 1997; Lahmy et al., 2013; Maurice et al., 1996; Meunier et al., 2006, 2013; Villard et al., 2009, 2011; Zussy et al., 2011, 2013). Therefore, the difference observed between Sc.Aβ-treated WT and Tc1 mice is most likely to result from the presence of the Hsa21 genes and is consistent with previously reported phenotypes of Tc1 mice (Ahmed et al., 2013; Gribble et al., 2013; Marechal et al., 2015; Morice et al., 2008; O’Doherty et al., 2005).

Although Tc1 mice showed a slightly lower alteration percentage or step-though latency than WT animals, there was no significant difference. This suggests that alteration of memory capacities in Tc1 mice begins at a young age but become firmly established at a later stage in this mouse model of DS. Previously, adult male Tc1 mice showed impaired spatial working memory, but unaffected long-term spatial reference memory in the water-maze (Morice et al., 2008). Similarly, Tc1 mice appeared selectively impaired for short-term memory but had intact long-term memory in the novel object recognition task (Morice et al., 2008; O’Doherty et al., 2005). These early memory deficits are consistent with the early intellectual disability observed in young DS people. These behavioural impairments were related to a deficit in early long-term potentiation, which might be explained by a reduced surface membrane expression of the α-amino-3-hydroxy-5-methyl-4-isoxazolepropionic acid receptor subunit GluR1 in *Tc1* mice (Morice et al., 2008). GluR5 subunit is also a possible candidate gene for the impaired memory observed in *Tc1* mice, as the *GRIK1* gene (which codes for GLUR5) maps to Hsa21 (Gregor et al., 1993). Indeed, GluR5 overexpression could alter the subunit composition and properties of heteromeric GluR-associated ion channels and could then have a detrimental effect on the short-term recognition memory in Tc1 mice (Morice et al., 2008). Of interest, reducing the genetic dosage of 13 conserved mouse genes located between *Abcg1* and *U2af1* in the telomeric part of Hsa21 in Tc1 mice rescued subtle impairments in reversal learning, working memory and did so partially in the rotarod test, but had no impact on hyperactivity or spatial learning. Therefore, these 13 genes were modifiers of Tc1-dependent memory and locomotor phenotypes (Marechal et al., 2015). Among them, phosphodiesterase 9A (PDE9A) appears as a relevant candidate to explain the Tc1 phenotype, and also Aβ toxicity. Inhibition of PDE9A successfully rescued learning impairments in AD mouse models and prevented Aβ-induced neurotoxicity (Kroker et al., 2014). Thus PDE9A overexpression in DS could be an exacerbating influence both for cognitive deficits in DS, as well as for AD in DS (Gardiner, 2014). Recently, Witton et al. (2015) reported dysfunctional connectivity between dentate gyrus and the CA3 pyramidal cell layer in Tc1 mice, demonstrating that ultrastructural abnormalities and impaired short-term plasticity at dentate gyrus–CA3 excitatory synapses culminate in impaired coding of new spatial information in CA3 and CA1 and disrupted behaviour in vivo.

Among the triplicated genes in Tc1 mice, DYRK1A expression seems to be an interesting gene candidate to explain the phenotype, and also the Aβ_25-35_-induced effects. As DYRK1A activity is correlated with its expression (Liu et al., 2008), Tc1 mice are likely to have an increase in DYRK1A activity. DYRK1A expression was enhanced by Aβ_25-35_ indicating an increase of DYRK1A activity. DYRK1A expression was slightly increased in the hippocampus and cortex of WT and Tc1 mice by the peptide, with Aβ_25-35_-treated Tc1 mice showing a +80% increase in the hippocampus and +120% increase in the cortex, as compared with the expression measured in Sc.Aβ-treated WT control mice. The protein therefore appeared to be highly reactive in the brain areas vulnerable to amyloid toxicity. Furthermore, DYRK1A expression could be directly modulated by Aβ oligomers, and could be also involved in the enhanced Aβ toxicity. A specific knockdown of DYRK1A expression by siRNA inhibited APP-induced neurodegeneration (Tahtouh et al., 2012) and, in cell cultures, a highly DYRK1A selective inhibitor, EHT 5372 (IC_50_=0.22 nM), prevented Aβ-induced Tau hyperphosphorylation (Coutadeur et al., 2015), demonstrating in vitro DYRK1A involvement in Aβ-induced toxicity. We have previously demonstrated the neuroprotective effects of the DYRK1A inhibitor L41 (IC_50_=10–60 nM) on Aβ_25-35_-induced toxicity in mouse brains, notably preventing the onset of memory impairments and of oxidative stress, both in terms of ROS accumulation and increased peroxidised lipids (Naert et al., 2015). Of note, Aβ_25-35_ had no effect on the level of oxidative stress in Tc1 mice, which already had a high level of oxidative stress.

Interestingly, the Tc1 mouse model is also trisomic for SOD1 (Ahmed et al., 2013; O’Doherty et al., 2005) and over-expression of SOD1 in the absence of a peroxide detoxifying enzyme in the same cellular compartment promotes oxidative stress (Usui et al., 2011). SOD1 triplication in Tc1 mice (Ahmed et al., 2013; O’Doherty et al., 2005) would then participate in the augmentation of oxidative stress observed in Tc1 mice and thus explaining the lack of Aβ_25-35_ effect.

In our study, in WT mice, Aβ_25-35_ induced a decrease in synaptophysin level, without any effect on expression of Arc, Egr1 and PSD95. In a previous study, Aβ_25-35_ decreased the expression of Arc, Egr1, PSD95 and synaptophysin in WT Swiss mice (Naert et al., 2015). This difference could be attributed to the two different strains of mice used, Swiss or 129S8×C57BL/6, since mouse backstrains present specific traits. Aβ_25-35_ was able to decrease the level of synaptophysin level in Tc1 mice more robustly. Moreover, Arc, Synaptophysin and, to a lesser extent, PSD95 were decreased in Tc1 mice. These lower levels in synaptic markers suggest that neurotoxicity is constitutively present in Tc1 mice that impacts synaptic physiology, thus explaining the important Aβ_25-35_-induced memory impairments. Indeed, the immediate early gene Arc is closely related to N-methyl-D-aspartate activity (Bloomer et al., 2008), and thus involved in memory processes in the hippocampus. PSD95 is critical for synaptic plasticity (Malinow and Malenka, 2002), particularly involved in recruiting and holding glutamate receptors at the membrane surface (Ango et al., 2000; Xiao et al., 1998). Synaptophysin is highly expressed in neurites and synaptic boutons and stabilises the synapse. The occurrence of memory impairment is correlated with a decline in these synaptic gene expression levels during aging (Blalock et al., 2003) and in an AD transgenic mice model (Dickey et al., 2003, 2004; Naert and Rivest, 2012). Tc1 mice present lower levels of synaptic markers resulting in memory impairments (Ma and Klann, 2011). So, although Aβ_25-35_ did not further alter the markers, alteration of brain plasticity observed in Tc1 mice may explain more drastic consequences of amyloid toxicity at the behavioural level, as compared to WT animals.

As observed in previous studies (Lahmy et al., 2013; Naert et al., 2015), Aβ_25-35_ increased GSK-3β activity in WT mice. Despite the high level of GSK-3β activity observed in Tc1 mice (our results, and Ahmed et al., 2013), Aβ_25-35_ was still able to enhance GSK-3β activity in the Tc1 mouse cortex. Although Aβ_25-35_ failed to enhance the phosphorylation of GSK-3β on Tyr^216^, suggesting that phosphorylation of Tyr^216^ had reached a maximum, Aβ_25-35_ decreased its phosphorylation on the inhibitory site Ser^9^. AKT phosphorylates GSK-3β on Ser^9^ (Fang et al., 2000). However, the reduced phosphorylation on Ser^9^ could not be caused by a decreased in AKT activity, since Sc.Aβ- and Aβ_25-35_-treated Tc1 mice exhibited a similar level of AKT phosphorylation on Ser^473^ both in the cortex and hippocampus. Many other protein kinases are able to phosphorylate GSK-3β on Ser^9^ (Fang et al., 2000; Medina et al., 2011; Medina and Wandosell, 2011) and could be involved in the Aβ_25-35_-induced reduction of GSK-3β phosphorylation on Ser^9^.

Although, no evidence supports GSK-3β phosphorylation at Tyr^216^ by DYRK1A or priming activity by DYRK1A on GSK-3β at Tyr^216^ or Ser^9^, we cannot exclude the possibility that the increase of Tyr^216^ phosphorylation resulted from an indirect effect of DYRK1A. DYRK1A directly phosphorylates GSK-3β on Thr^356^, thereby inhibiting GSK-3β activity (Song et al., 2015). In addition, the preferential DYRK1A inhibitor L41 prevented Aβ_25-35_-induced phosphorylation of GSK-3β on Tyr^216^ (Naert et al., 2015), suggesting that this Aβ_25-35_-induced increase of Tyr^216^ phosphorylation is a DYRK1A-dependent mechanism. Other kinases, such as CDK5, modulated directly phosphatases involved in Ser^9^ dephosphorylation (Morfini et al., 2004; Plattner et al., 2006). Although modulation of phosphatases by DYRK1A is not yet reported, this DYRK1A effect can be envisaged.

The expression and phosphorylation of AKT and GSK-3β have been previously studied in Tc1 mice (Ahmed et al., 2013; Sheppard et al., 2012). Some discrepancies exist and could be attributed to the different ages of the mice used, or to technical differences in these studies. Despite a transient increase of total AKT in two-month old Tc1 mice, at older ages no significant difference was observed (Ahmed et al., 2013; Sheppard et al., 2012). We observed a decrease of AKT phosphorylation at Ser^473^ with an unchanged level of total AKT both in cortex and hippocampus of Tc1 mice. Only a late increase of AKT phosphorylation was reported in the cortex of 20-month-old Tc1 mice (Ahmed et al., 2013; Sheppard et al., 2012), which is more consistent with previous data reported in other DS mouse models. An increase in AKT phosphorylation had been reported in Ts65Dn and Ts1Cje mice (Siarey et al., 2006; Siddiqui et al., 2006). This increase could be in part explained by the fact that SOD1 has been linked to increased AKT phosphorylation at Ser^473^ (Endo et al., 2007; Noshita et al., 2003). In our study, the decreased phosphorylation of AKT at Ser^473^ excludes SOD1 involvement and could rather result from AKT dephosphorylation by protein phosphatases.

As previously observed (Lahmy et al., 2013; Naert et al., 2015), Aβ_25-35_ injection activates pro-apoptotic pathways in both WT mice and Tc1 mice, despite already enhanced levels of apoptosis in Tc1 mice. In Tc1 mice, in addition to inducing pro-apoptotic protein expression, Aβ_25-35_ reduced anti-apoptotic proteins Bcl-W and Bcl-XL, enhancing apoptotic activity, since Bcl-XL translocates Bax from the mitochondria into the cytosol (Edlich et al., 2011). DYRK1A overexpression in Tc1 mice could participate in the enhanced apoptosis, since DYRK1A regulates pro-apoptotic pathways through regulation of GSK-3β activity. Indeed, GSK3 is able to directly enhance Bax activity in neuronal cells (Ngok-Ngam et al., 2013). A direct effect of oligomeric Aβ peptides could also be considered, as the peptides induced cell death through a Bax-dependent mechanism (Giovanni et al., 2000; Kudo et al., 2012). However, the mechanism involved in Aβ-induced Bax activation remains unknown. One potential explanation could be an Aβ-induced kinase activation, such as Aβ-induced activation of c-Jun N-terminal kinase (JNK). Aβ peptide also induced DYRK1A expression (Kimura et al., 2007), which is coupled to JNK1 activation. DYRK1A positively regulates apoptosis signal-regulating kinase 1 (ASK1)-mediated JNK1-signaling and appears to directly phosphorylate ASK1 (Choi and Chung, 2011). Therefore, DYRK1A is able to modulate apoptotic markers expression and activity and plays a key role in ASK1-mediated transmission of cell death signals.

## Conclusion

Tc1 mice carrying a freely segregating copy of Hsa21 and constituting a unique animal model of DS were used to study the influence of Hsa21 trisomy on Aβ toxicity in young mice. This in vivo study examined amyloid toxicity (Aβ_25-35_ toxicity) and learning and memory deficits in Tc1 mice. Tc1 mice have constitutively hallmarks of neurotoxicity, with oxidative stress, deficits in synaptic markers, activation of GSK-3β and increased expression of Bax and Bim). Injection of oligomeric Aβ_25-35_ peptide resulted in increased expression of pro-apoptotic markers and GSK-3β activity and resulted in more marked memory deficits than those observed in WT mice. However, several markers were not altered by the peptide injection, including ROS, synaptic deficits or the decrease in AKT activity. This study therefore showed that the developmental alterations in Tc1 mice where ~75% of Hsa21 genes are triplicated mask, or blunt, Aβ_25-35_ effects on numerous markers but facilitate its behavioural impact. *DYRK1A*, known to be especially involved in DS and Aβ toxicity, could be one of the genes involved in the neurotoxic effects of Aβ peptides. Alterations in the activity of other key regulators of Aβ pathology could also be involved in Aβ toxicity in the context of DS and explain the earlier onset and the prevalence of AD in DS subjects.
